# Azathioprine Has a Deleterious Effect on the Bone Health of Mice with DSS-Induced Inflammatory Bowel Disease

**DOI:** 10.3390/ijms20236085

**Published:** 2019-12-03

**Authors:** Stephanie Morgan, Kirsty M. Hooper, Elspeth M. Milne, Colin Farquharson, Craig Stevens, Katherine A. Staines

**Affiliations:** 1 School of Applied Sciences, Edinburgh Napier University, Edinburgh EH11 4BN, UK; 2 Babraham Institute, Cambridge CB22 3AT, UK; 3 The Royal (Dick) School of Veterinary Studies and The Roslin Institute, The University of Edinburgh, Edinburgh EH25 9RG, UK

**Keywords:** bone, inflammatory bowel disease, autophagy, colitis, osteoporosis, azathioprine

## Abstract

Patients with inflammatory bowel disease (IBD) often present poor bone health and are 40% more at risk of bone fracture. Studies have implicated autophagy in IBD pathology and drugs used to treat IBD stimulate autophagy in varying degrees, however, their effect on the skeleton is currently unknown. Here, we have utilised the dextran sulphate sodium (DSS) model of colitis in mice to examine the effects of the thiopurine drug azathioprine on the skeleton. Ten-week-old male mice (*n* = 6/group) received 3.0% DSS in their drinking water for four days, followed by a 14-day recovery period. Mice were treated with 10 mg/kg/day azathioprine or vehicle control. Histopathological analysis of the colon from DSS mice revealed significant increases in scores for inflammation severity, extent, and crypt damage (*p* < 0.05). Azathioprine provided partial protection to the colon, as reflected by a lack of significant difference in crypt damage and tissue regeneration with DSS treatment. MicroCT of vehicle-treated DSS mice revealed azathioprine treatment had a significant detrimental effect on the trabecular bone microarchitecture, independent of DSS treatment. Specifically, significant decreases were observed in bone volume/tissue volume (*p* < 0.01), and trabecular number (*p* < 0.05), with a concurrent significant increase in trabecular pattern factor (*p* < 0.01). Immunohistochemical labelling for LC3 revealed azathioprine to induce autophagy in the bone marrow. Together these data suggest that azathioprine treatment may have a deleterious effect on IBD patients who may already be at increased risk of osteoporotic bone fractures and thus will inform on future treatment strategies for patient stratification.

## 1. Introduction

Inflammatory bowel disease (IBD) is the name given to a group of conditions that affect the colon and the small intestine. The two main forms of IBD are ulcerative colitis and Crohn’s disease. Crohn’s disease can affect any part of the gastrointestinal tract and can cause transmural inflammation. In contrast, ulcerative colitis causes mucosal inflammation and is limited to the colon [1]. A recent review by NHS England revealed that the prevalence of IBD was 400 in every 100,000 people, which results in a total cost of £720 million a year in costs to the NHS [2].

Osteoporosis has been associated as secondary to a number of gastrointestinal conditions, including IBD [3,4]. Osteoporosis is a metabolic disease of the bone characterised by a reduction in bone mineral density and alterations in bone structure, which increases the likelihood of bone fracture. Significantly, patients with IBD often present with low bone mineral density (BMD), and are 40% more at risk of bone fracture than healthy individuals [3].

A number of risk factors exist for osteoporosis in IBD patients including malabsorption, chronic inflammation, low body mass index, use of glucocorticoids, vitamin D deficiency, and surgery [5]. Intestinal absorption of key determinants of bone health (e.g., calcium and vitamin D) is often compromised in IBD due to the reduction of intestinal mucosa [4]. Indeed, patients with IBD present with vitamin D [6,7] and calcium deficiencies [8]. IBD is also characterised by the chronic release of pro-inflammatory cytokines such as IL-6 and tumour necrosis factor-α (TNF-alpha)—the increased production of these cytokines can stimulate the receptor activator of nuclear κB (RANK)/RANK ligand (RANKL)/osteoprotegerin (OPG) axis and thus drive osteoclastic bone resorption [9,10,11]. Low body mass index is a well-established risk factor for low BMD and fracture, and studies have shown similar associations in patients with IBD [12]. However, other mechanisms may exist and the development of targeted therapies for bone loss in IBD requires a better understanding of the underlying cellular mechanisms.

Drugs used to treat IBD include glucocorticoids, immunosuppressants, aminosalicylates (5-ASA), and biologic agents. Risk of osteoporosis has been shown to be twice as high in IBD patients on corticosteroids [13], which is thought to be due to their effects on the RANK/RANKL/OPG axis, sex hormones, and calcium absorption [14]. Conversely, IBD patients on the monoclonal antibody infliximab have shown increases in their BMD and markers of bone turnover [15,16,17]. Similarly, patients on dual anti-TNF-α (infliximab or adalimumab) and azathioprine saw a significant positive effect on BMD at the lumbar spine [18]. There is an increasing demand to optimise existing medical therapies through patient stratification and personalised medicine [19]. We and others have previously shown that drugs currently used for treating IBD can affect the autophagy pathway [20]. Autophagy is an essential self-eating process that can degrade intracellular components such as organelles, foreign bodies, and insoluble protein aggregates through the formation and maturation of double membrane vesicles known as autophagosomes [21]. Specifically, we have shown that the thiopurine drug azathioprine is a potent inducer of autophagy in the colon, however its effects on bone health have yet to be established [22].

To directly examine this we have studied bones from a mouse model of IBD using dextran sulphate sodium (DSS)-induced colitis, which has been extensively used and validated by others to induce acute and chronic forms of inflammatory bowel disease. This model of colitis in mice is thought to involve the deterioration of the intestinal epithelial barrier through increased cell apoptosis, therefore allowing the influx of antigens and microorganisms and the subsequent increased expression of inflammatory markers [23,24]. Histologically, the colitis induced in this murine model exhibits a wide range of features similar to that seen in man [23]. Further, we and others have previously shown that DSS treatment causes detrimental effects on bone trabecular microarchitecture [25,26]. Here we have used the DSS model to examine the effects of azathioprine treatment on the skeleton in the context of IBD. The dose and duration of the DSS treatment, and the age, sex, and strain of the mice used were based on our previous study [26]. Male mice were also used to avoid any confounding actions of oestrogen on the skeleton. Data from this approach will help inform on personalised therapies for patients with poor bone health in IBD.

## 2. Results

### 2.1. Effect of Azathioprine on Body Weight in DSS Treated Mice

**Figure 1:**
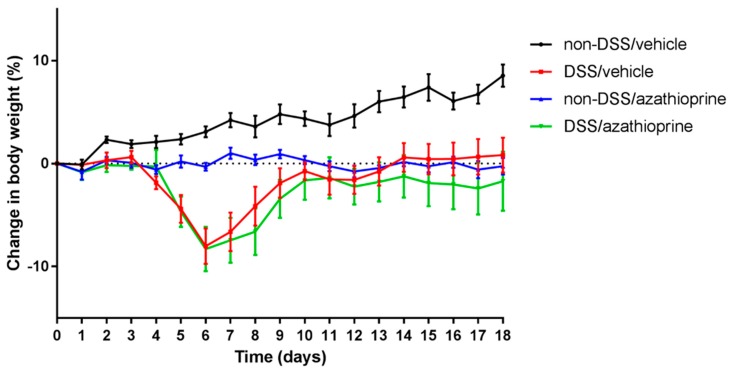
Body weight changes of azathioprine and vehicle treated mice treated with dextran sulphate sodium (DSS) followed by a recovery period. Percentage change in body weight of azathioprine and vehicle mice treated with or without 3% DSS for 4 days. Data are presented as mean ± S.E.M (*n* = 6/group).

To assess the effects of azathioprine on body weight of mice, colitis was induced with 3% DSS for 4 days in the presence of azathioprine (DSS/azathioprine) or vehicle control (DSS/vehicle). During the DSS treatment period (0–4 days), no significant weight loss was observed in any mouse treatment groups (Figure 1). Independent of azathioprine treatment, mice exhibited a rapid and significant weight loss from day 4 following DSS treatment (up to 7% in comparison to non-DSS treated mice, *p* < 0.05). Following this period of rapid weight loss, DSS/vehicle treated mice proceeded to gain weight until the end of the study. Weight gain was observed throughout the study period in the non-DSS/vehicle treated mice (Figure 1). In contrast, DSS/azathioprine treated mice exhibited a rapid and significant weight loss, followed by a brief period of weight gain, which plateaued from day 10 onwards (Figure 1). Non-DSS/azathioprine treated mice showed no significant weight gain throughout the experiment (Figure 1). Full details of the weight measurements and statistical significance over the 18-day treatment period is detailed in Supplementary Table S1.

### 2.2. Effect of Azathioprine on Colon Pathology in DSS Treated Mice

**Figure 2:**
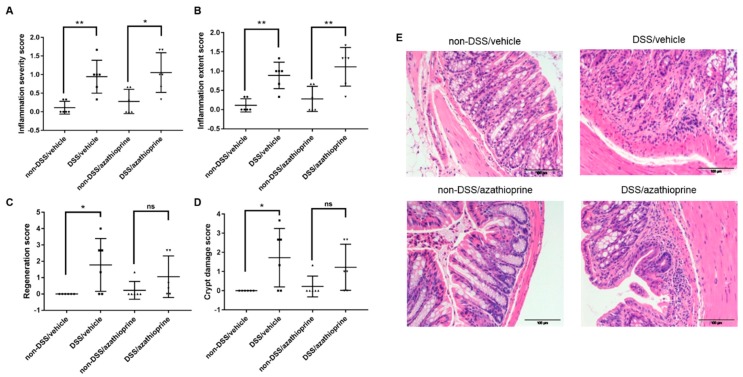
Colon pathology of azathioprine and vehicle treated mice treated with 3% DSS. Histological scoring of colons, (**A**) Inflammation severity score; (**B**) inflammation extent score; (**C**) regeneration score; (**D**) crypt damage score; (**E**) representative Hematoxylin & Eosin -stained sections of colon. Data are presented as mean ± S.D. (*n* = 6/group). * *p* < 0.05, ** *p* < 0.01. Scale bar = 100 µm.

To assess the effects of DSS on mucosal integrity, detailed histological analysis was performed on the colon from control and DSS/azathioprine or DSS/vehicle mice. Histological scores for all parameters were minimal in the non-DSS treated mice, and there were no notable differences observed with azathioprine treatment in this group (Figure 2). In contrast, histological analysis of the colon from DSS mice revealed significant increases in scores for inflammation severity (Figure 2A, *p* < 0.05) and extent (Figure 2B, *p* < 0.01), consistent with previous studies and indicative of successful induction of colitis. It was also observed that the colons from DSS/vehicle mice showed decreased tissue regeneration (as indicated by the higher regeneration score; Figure 2C, *p* < 0.05) and increased crypt damage (Figure 2D, *p* < 0.05) in comparison with the non-DSS/vehicle mice.

No significant differences were observed in tissue regeneration (Figure 2C) and crypt damage (Figure 2D) in non-DSS/azathioprine and DSS/azathioprine treated mice, indicative of a partial protection of azathioprine treatment to the colon. Regional specific changes in the parameters examined were also observed, with significant pathology localised to the distal aspect of the colon (Figure S1).

### 2.3. Effect of Azathioprine on Bone Phenotype in DSS Treated Mice

**Figure 3:**
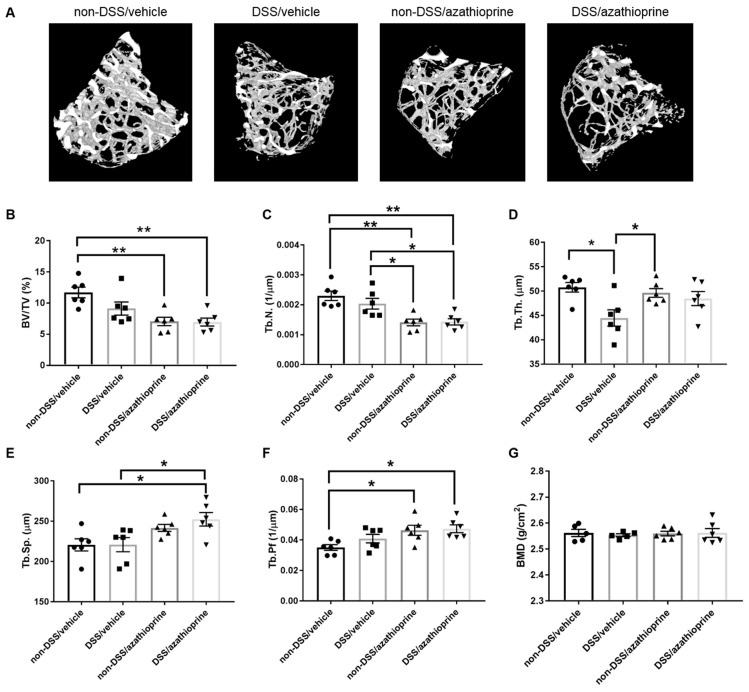
Trabecular bone microarchitecture of azathioprine and vehicle treated mice treated with 3% DSS. (**A**) Representative 3D microCT reconstructions. Trabecular bone parameters between treated and control groups, (**B**) bone volume/tissue volume (BV/TV), (**C**) trabecular number (Tb. N.), (**D**) trabecular thickness (Tb. Th.), (**E**) trabecular separation (Tb. Sp.), (**F**) trabecular pattern factor (Tb. Pf.), (**G**) trabecular bone mineral density (BMD). Data are presented as mean ± S.D. (*n* = 6/group). * *p* < 0.05. ** *p* < 0.01.

DSS-treated mice showed worsened trabecular microarchitecture compared with non-DSS treated mice as demonstrated by micro computed-tomography (CT) (Figure 3A). Specifically, DSS-treated mice exhibited a significant decrease in trabecular thickness (Figure 3D, *p* < 0.05). Non-significant decreases in bone volume/tissue volume (BV/TV) (Figure 3B), and trabecular number (Figure 3C), and increases in trabecular separation (Figure 3E) and pattern factor (Figure 3F) were also observed in DSS-treated mice. Treatment with azathioprine alone had a significant detrimental effect on the trabecular bone microarchitecture, independent of DSS treatment. Indeed, significant decreases were observed in BV/TV (Figure 3B, *p* < 0.01), and trabecular number (Figure 3C, *p* < 0.01), with a concurrent significant increase in trabecular pattern factor (Figure 3F, *p* < 0.05) indicative of a more disorganised trabecular structure. No effects of DSS or azathioprine treatment were observed on trabecular BMD (Figure 3G).

### 2.4. Induction of Autophagy by Azathioprine in DSS Treated Mice

**Figure 4:**
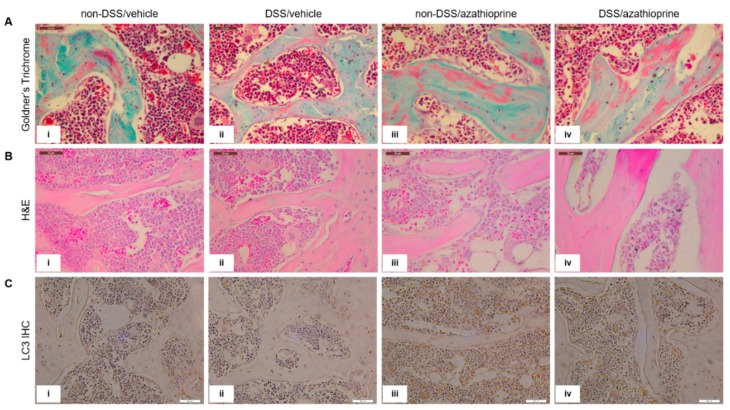
Histological staining and immmunohistochemical labelling of tibia trabecular bone in sections of the tibia. (**A**) Goldner’s Trichrome, (**B**) H&E, (**C**) LC3 immunolabelling. LC3-positive immunolabelling is presented as brown staining. (i) non-DSS/vehicle; (ii) DSS/vehicle; (iii) non-DSS/azathioprine; (iv) DSS/azathioprine. Scale bar = 50 µm. Images are representative of 4 different mice/group.

Histological analysis of the tibia sections appeared to confirm the results from the microCT analysis, with an apparent reduction in bone volume (indicated by increased red osteoid staining) in the trabecular bone in both the DSS treated mice (Figure 4Aii, in comparison to Figure 4Ai) and those treated with azathioprine (Figure 4Aiii,iv, in comparison to Figure 4Ai). To examine whether azathioprine affects autophagy activity in the skeleton of mice, immunohistochemistry for the autophagy marker LC3 was conducted. LC3 labelling was observed in the osteoblasts lining the trabecular bone, in the osteocytes and throughout the bone marrow. No differences were observed between non-DSS and DSS treated mice (Figure 4Ci, in comparison to Figure 4Cii). However, azathioprine treatment appeared to modestly increase the intensity of LC3 labelling, independent of DSS treatment and in particular within the bone marrow, although no significant differences were observed upon quantification of immunolabelling intensity (Figure 4Ciii,iv, in comparison with Figure 4Ci,ii, Figure S2).

## 3. Discussion

Patients with IBD often present poor bone health and are 40% more at risk of bone fracture [3]. Azathioprine is widely used in the treatment of IBD and has been proven to be highly effective, however it has previously been linked to an increase in fracture risk in humans [27]. Here we utilised the DSS model of colitis in mice to delineate the effects of the azathioprine on the skeleton. Histopathological analysis of the colon revealed successful induction of colitis in DSS treated mice, however, it also revealed no differences in the severity or extent of inflammation in azathioprine treated mice compared to vehicle-treated mice. This suggests that any noted effects of azathioprine on the skeleton may be direct and not a consequence of altered nutrition through malabsorption of nutrients.

There are a number of risk factors associated with IBD-related bone loss including poor nutrient intake/absorption, chronic inflammation, and use of glucocorticoids [5]. Central to the inflammatory response is the chronic release of the pro-inflammatory cytokines IL-6 and TNF-α. Indeed IL-6 has been identified as the predominant cytokine mediating the bone abnormalities, and genetic variations in IL-6 correlate well with the clinical course of IBD and the extent of bone loss [28,29]. These pro-inflammatory cytokines are known to promote bone loss directly, but also through altered sensitivity and secretion of growth hormone and insulin-like growth factor in IBD [30,31,32]. DSS-induced colitis is the result of deterioration of the epithelial barrier, allowing for the influx of antigens and microorganisms, and prompting the increased expression of these pro-inflammatory mediators [23,24]. Indeed this has previously been shown to have detrimental effects on bone quality [25,26]. In accordance with these studies, we observed worsened bone trabecular microarchitecture with DSS treatment in our mice. Further, when azathioprine was administered alone it was also found to have a detrimental effect on bone microarchitecture. This is consistent with previous reports of increased overall skeletal fracture risk in individuals prescribed azathioprine [27], although it contradicts those in which combination of anti-TNF-α and azathioprine had a positive effect [18]. The anti-TNF-α monoclonal antibody infliximab has on its own been associated with increases in BMD and markers of bone which may provide explanation as to the disparity between these results [15,16,17]. In addition, it has been suggested that azathioprine can disrupt the bone remodelling process in a rat model by suppressing T lymphocytes causing disturbances in the RANKL system responsible for osteoclast formation and activity [33]. Specifically, the authors found that although the length and diameter of the bones remained unchanged, azathioprine caused an overall reduction in femur and tibia mass, whilst also reducing the calcium content. Further, the thickness of the trabeculae in the femur was found to be reduced in rats when treated with azathioprine in both the distal epiphysis and metaphysis [33]. The bisphosphonate alendronate prevented the development of these skeletal changes when administered in combination with azathioprine [33]. These findings complement our findings here that the administration of azathioprine may contribute to overall bone loss and trabecular bone deterioration. This suggests that azathioprine alone may therefore not be a suitable drug of choice for IBD patients who are more at risk of osteoporotic bone fractures, such as the elderly.

We hypothesised that the detrimental effects on bone caused by azathioprine may result in the induction of the autophagy pathway, as azathioprine has already been shown to induce this process in peripheral blood mononuclear cells and colorectal cancer cells [22,34]. Autophagy is a homeostatic process in which cells degrade protein aggregates and damaged organelles [35]. Upon the induction of autophagy, LC3-I becomes lipidated and becomes LC3-II before inserting into the autophagosome membrane [36]. Because of this, the detection of LC3 within a sample is a recognised marker used to show the presence of autophagy. Here we revealed increased LC3 labelling in our azathioprine treated mice, suggestive of autophagy induction. This suggests that azathioprine is an effective inducer of autophagy activity in the skeleton. The reasons for this are currently speculative, as it is currently not known whether autophagy is indirectly induced as a survival mechanism to cope with the adverse effects caused by azathioprine on bone health. Similarly, the precise effects of azathioprine on osteoblast function are currently unknown. The effects of another autophagy inducer, rapamycin, are well documented however, albeit through somewhat controversial findings. It has been shown that rapamycin, in the presence of lipopolysaccharides, can promote the differentiation of human embryonic stem cells (hESCs) into mature osteoblasts by modulating mTOR signalling [37,38]. However, it was also found that rapamycin inhibits osteoblast proliferation and differentiation in MC3T3-E1 cells. It was observed that even at low concentrations (0.1–20 nM), rapamycin reduced osteocalcin and osterix mRNA expression in differentiating MC3T3-E1 osteoblasts, as well as reducing their mineralisation capacity [39]. Therefore, this further highlights the need to understand more fully the mechanism of action of azathioprine before a better level of care for patients can be provided.

In conclusion, the data in this manuscript suggest that azathioprine treatment may have a deleterious effect on bone health in IBD patients who may already be at increased risk of osteoporotic bone fractures, and thus will inform on future treatment strategies for patient stratification.

## 4. Materials and Methods

### 4.1. Animals

Male 10-week old C57BL/6J mice (*n* = 6/group) (Charles River, UK) were treated with 3% DSS (molecular mass ~40,000 kD; MP Biomedical, Eschwege, Germany) in their drinking water ad libitum for 4 days, following which they were given normal tap water for a 14-day recovery period. Control (non-DSS treated) male mice received normal tap water for the duration of the study. The dose and duration of the DSS treatment was based on previous studies using the same mouse strain, age, and sex [26]. It is important to note that this model is unable to distinguish between ulcerative colitis and Crohn’s disease. Mice were treated using an oral gavage daily throughout the experiment with 10 mg/kg/day of azathioprine or a vehicle control (*n* = 6/group). The health status of the DSS-treated mice was scored daily, with particular attention paid to their coat condition, mobility, presence of blood in stools, and eye clarity. The body weights of all mice were recorded daily. After the 14-day recovery period, the mice were culled and blood, colon, and bone samples collected. Mice were kept in polypropylene cages, with light/dark 12 h cycles, at 21 ± 2 °C, and fed ad libitum with a maintenance diet (Special Diet Services, Witham, UK). All experimental protocols were approved by Roslin Institute’s Animal Users Committee (A911; 3/10/2017) and the animals were maintained in accordance with UK Home Office guidelines for the care and use of laboratory animals.

### 4.2. Colon Pathology

The colon was dissected from all mice, measured and fixed in 4% paraformaldehyde (PFA) for 24 h. Each colon was divided into 3 transverse segments including proximal, middle, and distal portions. Tissue processing, wax embedding, sectioning (5 µm thick) and Hematoxylin and eosin (H&E) staining were conducted following routine procedures. Colon pathology was graded blind on sections from all 3 segments of each mouse using an established histological grading scheme (Table S2) [40,41]. Scores from all three segments were averaged to provide an overall pathology score, as well being analysed in the separate regions of the colon.

### 4.3. Bone Histology and Immunohistochemistry

The tibia was dissected from all mice, fixed in 4% formaldehyde, and decalcified in 10% ethylenediaminetetraacetic acid (EDTA) at 4 °C. Tissue processing, wax embedding, and sectioning (5 µm thick) were done following routine procedures. Tibia sections were stained for histological analysis using the Goldner’s stain, and H&E following standard protocols. For immunohistochemistry, sections were dewaxed in xylene, rehydrated in graded alcohol, and incubated for 90 min at 70 °C in 10 mM citrate buffer for antigen retrieval. Any endogenous peroxidase activity was blocked by using 0.3% H_2_O_2_ for 30 min at room temperature. LC3 (1:500, polyclonal raised in rabbit; MBL Bio) antibodies were used with an appropriate IgG control (Suppl. Figure 2). The Vectastain ABC universal kit (Vector Laboratories, Peterborough, UK) was used according to the manufacturer’s instructions. The samples were counterstained with haematoxylin before being dehydrated and mounted using DePex. Quantification of immunolabelling was conducted using reciprocal intensity and the open source software Image J (http://fiji.sc/) [42].

### 4.4. MicroCT

To evaluate trabecular microarchitecture and cortical bone geometry of the tibia from control and DSS-treated mice, we used microCT (Skyscan 1172 X-ray microtomograph, Bruker, Kontich, Belgium) as described previously [43]. In brief, high-resolution scans with an isotropic voxel size of 5 µm were acquired (60 kV, 0.5 mm aluminium filter, 0.6° rotation angle). Two images were averaged at each rotation angle. Scan reconstruction was conducted using NRecon software (Bruker) and each bone was analysed using CtAN (Bruker). For the trabecular analysis, the base of the growth plate was used as a standard reference point. A 1.25 mm trabecular bone region, located at 5% of the total length beneath this reference point, was analysed. To investigate the changes in the cortical bone geometry, two 0.5 mm sections were analysed at 37% and 50% of the total bone length from the reference starting slice (first appearance of the medial tibial condyles). To assess BMD, phantoms were used to calibrate the CTAn software. BMD phantoms of known calcium hydroxyapatite mineral densities of 0.25 and 0.75 g/cm^3^ were scanned and reconstructed using the same parameters as used for bone samples.

### 4.5. Statistical Analysis

Data are expressed as the mean ± standard deviation (S.D.). A power analysis was conducted on microCT data from a previous study using an identical model and 6 experimental mice per group were required to detect statistically significant differences in the bone trabecular microarchitecture [26]. Analysis was performed by one-way analysis of variance (ANOVA) with appropriate post-hoc tests. *p* < 0.05 was considered to be significant and noted as *, with *p* values of < 0.01 and < 0.001 were noted as ** and *** respectively.

## Supplementary Materials

Supplementary materials can be found at https://www.mdpi.com/1422-0067/20/23/6085/s1.

## References

[1] Zhang Y.-Z., Li Y.-Y. (2014). Inflammatory bowel disease: Pathogenesis. World J. Gastroenterol..

[2] England N. (2013). Nhs Standard Contract for Colorectal: Complex Inflammatory Bowel Disease (Adult). NHS Engl..

[3] Ali T., Lam D., Bronze M.S., Humphrey M.B. (2009). Osteoporosis in Inflammatory Bowel Disease. Am. J. Med..

[4] Bianchi M.L. (2010). Inflammatory bowel diseases, celiac disease, and bone. Arch. Biochem. Biophys..

[5] Lima C.A. (2015). Risk factors for osteoporosis in inflammatory bowel disease patients. World J. Gastrointest. Pathophysiol..

[6] Blanck S., Aberra F. (2013). Vitamin D deficiency is associated with ulcerative colitis disease activity. Dig. Dis. Sci..

[7] Jørgensen S.P., Hvas C.L., Agnholt J., Christensen L.A., Heickendorff L., Dahlerup J.F. (2013). Active Crohn’s disease is associated with low vitamin D levels. J. Crohns Colitis.

[8] Silvennoinen J., Lamberg-Allardt C., Kärkkäinen M., Niemelä S., Lehtola J. (1996). Dietary calcium intake and its relation to bone mineral density in patients with inflammatory bowel disease. J. Intern. Med..

[9] Moschen A.R., Kaser A., Enrich B., Ludwiczek O., Gabriel M., Obrist P., Wolf A.M., Tilg H. (2005). The RANKL/OPG system is activated in inflammatory bowel diseases and relates to the state or bone loss. Gut.

[10] Bernstein C.N., Sargent M., Leslie W.D. (2005). Serum osteoprotegerin is increased in Crohn’s disease: A population-based case control study. Inflamm. Bowel Dis..

[11] Turk N., Cukovic-Cavka S., Korsic M., Turk Z., Vucelic B. (2009). Proinflammatory cytokines and receptor activator of nuclear factor κB-ligand/osteoprotegerin associated with bone deterioration in patients with Crohn’s disease. Eur. J. Gastroenterol. Hepatol..

[12] Lee N., Radford-Smith G.L., Forwood M., Wong J., Taaffe D.R. (2009). Body composition and muscle strength as predictors of bone mineral density in Crohn’s disease. J. Bone Miner. Metab..

[13] Abraham B.P., Prasad P., Malaty H.M. (2014). Vitamin D deficiency and corticosteroid use are risk factors for low bone mineral density in inflammatory bowel disease patients. Dig. Dis. Sci..

[14] Lane N.E. (2019). Glucocorticoid-Induced Osteoporosis: New Insights into the Pathophysiology and Treatments. Curr. Osteoporos. Rep..

[15] Miheller P., Muzes G., Rácz K., Blázovits A., Lakatos P., Herszényi L., Tulassay Z. (2007). Changes of OPG and RANKL concentrations in Crohn’s disease after infliximab therapy. Inflamm. Bowel Dis..

[16] Franchimont N., Putzeys V., Collette J., Vermeire S., Rutgeerts P., De Vos M., Van Gossum A., Franchimont D., Fiasse R., Pelckmansj P. (2004). Rapid improvement of bone metabolism after infliximab treatment in Crohn’s disease. Aliment. Pharmacol. Ther..

[17] Abreu M.T., Geller J.L., Vasiliauskas E.A., Kam L.Y., Vora P., Martyak L.A., Yang H., Hu B., Lin Y.C., Keenan G. (2006). Treatment with infliximab is associated with increased markers of bone formation in patients with Crohn’s disease. J. Clin. Gastroenterol..

[18] Krajcovicova A., Hlavaty T., Killinger Z., Miznerova E., Toth J., Letkovsky J., Nevidanska M., Cierny D., Koller T., Zelinkova Z. (2014). Combination therapy with an immunomodulator and anti-TNFα agent improves bone mineral density in IBD patients. J. Crohns Colitis.

[19] Denson L.A. (2013). The role of the innate and adaptive immune system in pediatric inflammatory bowel disease. Inflamm. Bowel Dis..

[20] Hooper K.M., Barlow P.G., Stevens C., Henderson P. (2017). Inflammatory bowel disease drugs: A focus on autophagy. J. Crohns Colitis.

[21] Yu L., Chen Y., Tooze S.A. (2018). Autophagy pathway: Cellular and molecular mechanisms. Autophagy.

[22] Hooper K.M., Casanova V., Kemp S., Staines K.A., Satsangi J., Barlow P.G., Henderson P., Stevens C. (2019). The Inflammatory Bowel Disease Drug Azathioprine Induces Autophagy via mTORC1 and the Unfolded Protein Response Sensor PERK. Inflamm. Bowel Dis..

[23] Perše M., Cerar A. (2012). Dextran sodium sulphate colitis mouse model: Traps and tricks. J. Biomed. Biotechnol..

[24] Araki Y., Mukaisyo K.I., Sugihara H., Fujiyama Y., Hattori T. (2010). Increased apoptosis and decreased proliferation of colonic epithelium in dextran sulfate sodium-induced colitis in mice. Oncol. Rep..

[25] Hamdani G., Gabet Y., Rachmilewitz D., Karmeli F., Bab I., Dresner-Pollak R. (2008). Dextran sodium sulfate-induced colitis causes rapid bone loss in mice. Bone.

[26] Dobie R., MacRae V.E., Pass C., Milne E.M., Ahmed S.F., Farquharson C. (2018). Suppressor of cytokine signaling 2 (Socs2) deletion protects bone health of mice with DSS-induced inflammatory bowel disease. Dis. Model. Mech..

[27] Vestergaard P., Rejnmark L., Mosekilde L. (2006). Methotrexate, azathioprine, cyclosporine, and risk of fracture. Calcif. Tissue Int..

[28] Schulte C.M.S., Dignass A.U., Goebell H., Roher H.D., Schulte K.M. (2000). Genetic factors determine extent of bone loss in inflammatory bowel disease. Gastroenterology.

[29] Paganelli M., Albanese C., Borrelli O., Civitelli F., Canitano N., Viola F., Passariello R., Cucchiara S. (2007). Inflammation is the main determinant of low bone mineral density in pediatric inflammatory bowel disease. Inflamm. Bowel Dis..

[30] Fernandez-Vojvodich P., Zaman F., Sävendahl L. (2013). Interleukin-6 acts locally on the growth plate to impair bone growth. Ann. Rheum. Dis..

[31] Wong S., Smyth A., McNeill E., Galloway P., Hassan K., McGrogan P., Ahmed S. (2010). The growth hormone insulin‐like growth factor 1 axis in children and adolescents with inflammatory bowel disease and growth retardation. Clin. Endocrinol..

[32] Wong S.C., Dobie R., Altowati M.A., Werther G.A., Farquharson C., Ahmed S.F. (2016). Growth and the growth hormone-insulin like growth factor 1 axis in children with chronic inflammation: Current Evidence, Gaps in Knowledge, and Future Directions. Endocr. Rev..

[33] Cegiela U., Kaczmarczyk-Sedlak I., Pytlik M., Folwarczna J., Nowińska B., Fronczek-Sokół J. (2013). Alendronate prevents development of the skeletal changes induced by azathioprine in rats. Acta Pol. Pharm. Drug Res..

[34] Chaabane W., Appell M.L. (2016). Interconnections between apoptotic and autophagic pathways during thiopurine-induced toxicity in cancer cells: The role of reactive oxygen species. Oncotarget.

[35] Henderson P., Stevens C. (2012). The Role of Autophagy in Crohn’s Disease. Cells.

[36] Nollet M., Santucci-Darmanin S., Breuil V., Al-Sahlanee R., Cros C., Topi M., Momier D., Samson M., Pagnotta S., Cailleteau L. (2014). Autophagy in osteoblasts is involved in mineralization and bone homeostasis. Autophagy.

[37] Lee K.-W., Yook J.-Y., Son M.-Y., Kim M.-J., Koo D.-B., Han Y.-M., Cho Y.S. (2010). Rapamycin promotes the osteoblastic differentiation of human embryonic stem cells by blocking the mTOR pathway and stimulating the BMP/Smad pathway. Stem Cells Dev..

[38] Li X., Chang B., Wang B., Bu W., Zhao L., Liu J., Meng L., Wang L., Xin Y., Wang D. (2017). Rapamycin promotes osteogenesis under inflammatory conditions. Mol. Med. Rep..

[39] Singha U.K., Jiang Y., Yu S., Luo M., Lu Y., Zhang J., Xiao G. (2008). Rapamycin inhibits osteoblast proliferation and differentiation in MC3T3-E1 cells and primary mouse bone marrow stromal cells. J. Cell. Biochem..

[40] Williams K.L., Fuller C.R., Dieleman L.A., DaCosta C.M., Haldeman K.M., Sartor R.B., Lund P.K. (2001). Enhanced survival and mucosal repair after dextran sodium sulfate-induced colitis in transgenic mice that overexpress growth hormone. Gastroenterology.

[41] Dieleman L.A., Palmen M.J.H.J., Akol H., Bloemena E., Peña A.S., Meuwissen S.G.M., Van Rees E.P. (1998). Chronic experimental colitis induced by dextran sulphate sodium (DSS) is characterized by Th1 and Th2 cytokines. Clin. Exp. Immunol..

[42] Nguyen D.H., Zhou T., Shu J., Mao J.-H. Quantifying Chromogen Intensity in Immunohistochemistry via Reciprocal Intensity. www.CancerInCytes.org.

[43] Staines K.A., Javaheri B., Hohenstein P., Fleming R., Ikpegbu E., Unger E., Hopkinson M., Buttle D.J., Pitsillides A.A., Farquharson C. (2017). Hypomorphic conditional deletion of E11/Podoplanin reveals a role in osteocyte dendrite elongation. J. Cell. Physiol..

