# Supplementary material for: Azathioprine Has a Deleterious Effect on the Bone Health of Mice with DSS-Induced Inflammatory Bowel Disease
Source: Int J Mol Sci. 2019 Dec 3;20(23):6085. doi: 10.3390/ijms20236085 (PMC6929096; doi:10.3390/ijms20236085)
Supplement: Supplementary file 1 [file ijms-20-06085-s001.pdf]

	Groups	Significance	P value
Day 5	non-DSS/vehicle vs. DSS/vehicle	**	0.0011
	non-DSS/vehicle vs. DSS/azathioprine	***	0.0006
	non-DSS/azathioprine vs. DSS/azathioprine	*	0.0384
Day 6	non-DSS/vehicle vs. DSS/vehicle	****	<0.0001
	non-DSS/vehicle vs. DSS/azathioprine	****	<0.0001
	DSS/vehicle vs. non-DSS/azathioprine	***	0.0001
	non-DSS/azathioprine vs. DSS/azathioprine	****	<0.0001
Day 7	non-DSS/vehicle vs. DSS/vehicle	****	<0.0001
	non-DSS/vehicle vs. DSS/azathioprine	****	<0.0001
	DSS/vehicle vs. non-DSS/azathioprine	***	0.0001
	non-DSS/azathioprine vs. DSS/azathioprine	****	<0.0001
Day 8	non-DSS/vehicle vs. DSS/vehicle	***	0.0001
	non-DSS/vehicle vs. DSS/azathioprine	****	<0.0001
	non-DSS/azathioprine vs. DSS/azathioprine	***	0.0006
Day 9	non-DSS/vehicle vs. DSS/vehicle	**	0.0013
	non-DSS/vehicle vs. DSS/azathioprine	****	<0.0001
Day 10	non-DSS/vehicle vs. DSS/vehicle	*	0.0267
	non-DSS/vehicle vs. DSS/azathioprine	**	0.0051
Day 11	non-DSS/vehicle vs. DSS/vehicle	*	0.0195
	non-DSS/vehicle vs. DSS/azathioprine	*	0.0256
Day 12	non-DSS/vehicle vs. DSS/vehicle	**	0.0033
	non-DSS/vehicle vs. non-DSS/azathioprine	*	0.0158
	non-DSS/vehicle vs. DSS/azathioprine	***	0.0008
Day 13	non-DSS/vehicle vs. DSS/vehicle	***	0.001
	non-DSS/vehicle vs. non-DSS/azathioprine	**	0.002
	non-DSS/vehicle vs. DSS/azathioprine	****	<0.0001
Day 14	non-DSS/vehicle vs. DSS/vehicle	**	0.0067
	non-DSS/vehicle vs. non-DSS/azathioprine	**	0.0028
	non-DSS/vehicle vs. DSS/azathioprine	***	0.0001
Day 15	non-DSS/vehicle vs. DSS/vehicle	***	0.0007
	non-DSS/vehicle vs. non-DSS/azathioprine	***	0.0001
	non-DSS/vehicle vs. DSS/azathioprine	****	<0.0001
Day 16	non-DSS/vehicle vs. DSS/vehicle	*	0.0105
	non-DSS/vehicle vs. non-DSS/azathioprine	**	0.0059
	non-DSS/vehicle vs. DSS/azathioprine	****	<0.0001
Day 17	non-DSS/vehicle vs. DSS/vehicle	**	0.0045
	non-DSS/vehicle vs. non-DSS/azathioprine	***	0.0003
	non-DSS/vehicle vs. DSS/azathioprine	****	<0.0001
Day 18	non-DSS/vehicle vs. DSS/vehicle	***	0.0001
	non-DSS/vehicle vs. non-DSS/azathioprine	****	<0.0001
	non-DSS/vehicle vs. DSS/azathioprine	****	<0.0001

Suppl. Table S1: Significance of weight changes between treatment groups of mice in DSS-study taken daily over the 18-day treatment period.

**Inflammation severity**	0 – none; 1 – mild; 2 – moderate; 3 - severe
**Inflammation extent**	0 – none; 1 – mucosa; 2 – mucosa and submucosa; 3 - transmural
**Crypt damage**	0 – none; 1 – basal 1/3; 2 – basal 2/3; 3 – crypt loss, surface epithelium present; 4 – crypts and surface epithelium lost
**Regeneration**	0 – complete tissue regeneration or normal tissue; 1 – almost complete regeneration; 2 – regeneration with crypt depletion; 3 – surface epithelium not intact; 4 – no tissue repair

Suppl. Table S2: Scoring criteria of colon pathology.

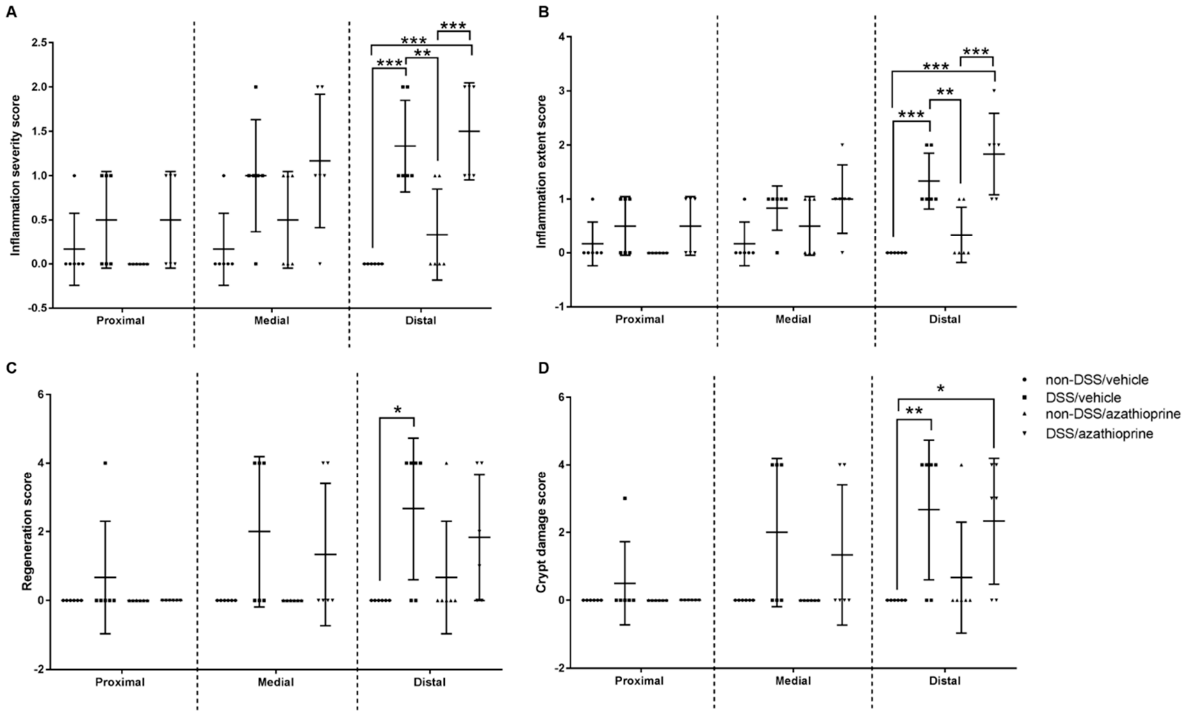

Suppl. Fig. S1: Colon pathology of azathioprine and vehicle treated mice treated with 3% DSS. Histological scoring of proximal, middle and distal regions of the colon (A) Inflammation severity score (B) Inflammation extent score (C) Regeneration score (D) Crypt damage score. Data are presented as mean ± S.D. (n=6/group). $P<0.05$*, $P<0.01$**, $P<0.001$***.

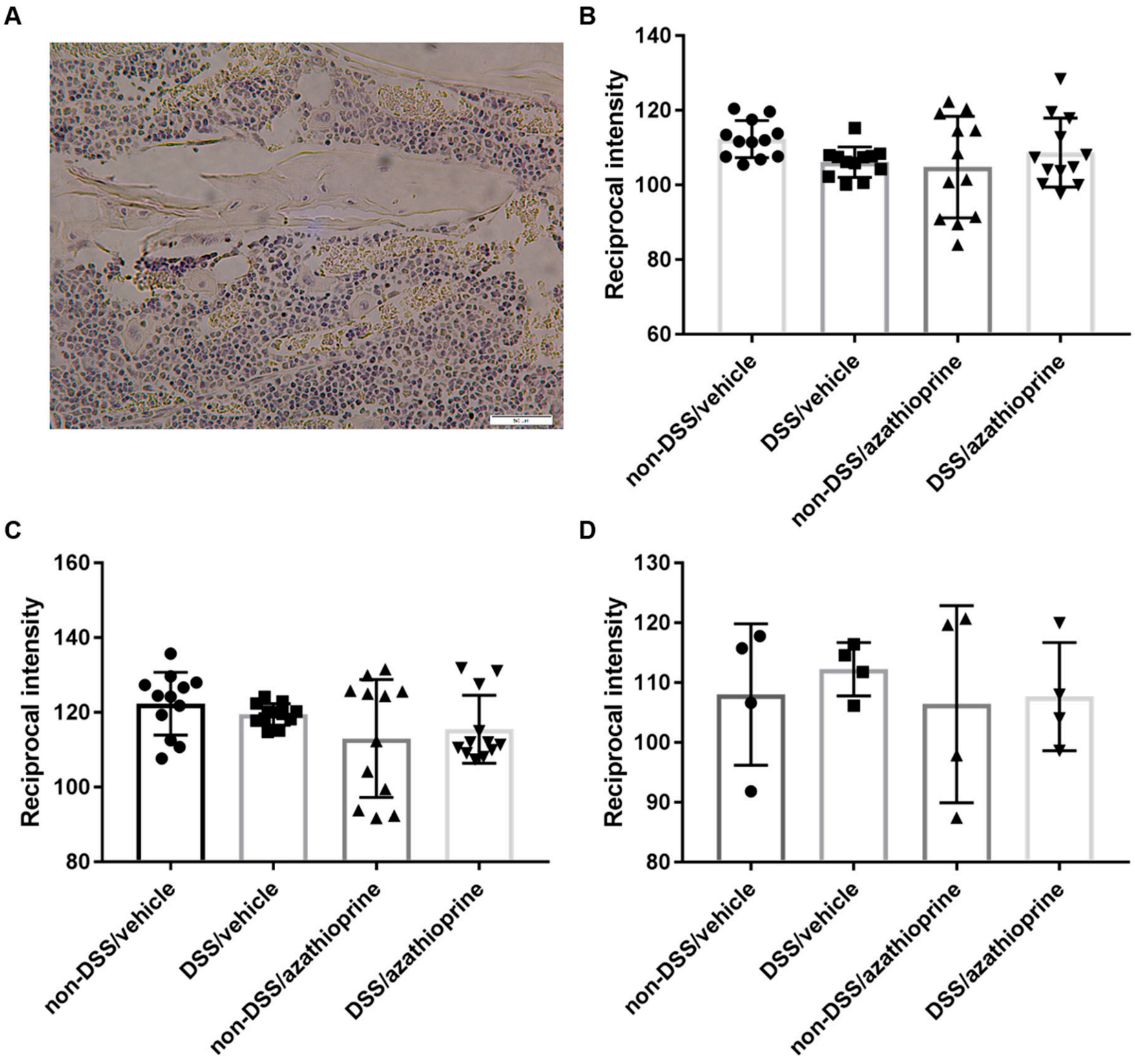

Suppl. Fig. S2: (A) Appropriate IgG control for LC3 immunolabelling. Scale bar = 50μm. Quantification of immunohistochemistry – reciprocal intensity of (B) osteoblasts (C) bone marrow (D) whole bone slice. Data are presented as mean ± S.D.
